# Immanent conditions determine imminent collapses: nutrient regimes define the resilience of macroalgal communities

**DOI:** 10.1098/rspb.2016.2814

**Published:** 2017-03-22

**Authors:** Jordi Boada, Rohan Arthur, David Alonso, Jordi F. Pagès, Albert Pessarrodona, Silvia Oliva, Giulia Ceccherelli, Luigi Piazzi, Javier Romero, Teresa Alcoverro

**Affiliations:** 1 Centre d'Estudis Avançats de Blanes (CEAB-CSIC), Carrer d'Accés a la cala Sant Francesc 14, 17300 Blanes, Spain; 2 Nature Conservation Foundation, 3076/5, 4th Cross, Gokulam Park, 570002 Mysore, Karnataka, India; 3 School of Ocean Sciences, Bangor University, Menai Bridge, Wales LL59 5AB, UK; 4 Dipartimento di Scienze della Natura e del Territorio (DIPNET), Università di Sassari, Via Piandanna 4, Sassari, Italy; 5 Departament d'Ecologia, Facultat de Biologia, Universitat de Barcelona, Avenue Diagonal 643, 08028 Barcelona, Spain

**Keywords:** alternative stable states, catastrophic shifts, buffer capacity, tipping points, sea urchin barrens, compensatory feeding

## Abstract

Predicting where state-changing thresholds lie can be inherently complex in ecosystems characterized by nonlinear dynamics. Unpacking the mechanisms underlying these transitions can help considerably reduce this unpredictability. We used empirical observations, field and laboratory experiments, and mathematical models to examine how differences in nutrient regimes mediate the capacity of macrophyte communities to sustain sea urchin grazing. In relatively nutrient-rich conditions, macrophyte systems were more resilient to grazing, shifting to barrens beyond 1 800 g m^−2^ (urchin biomass), more than twice the threshold of nutrient-poor conditions. The mechanisms driving these differences are linked to how nutrients mediate urchin foraging and algal growth: controlled experiments showed that low-nutrient regimes trigger compensatory feeding and reduce plant growth, mechanisms supported by our consumer–resource model. These mechanisms act together to halve macrophyte community resilience. Our study demonstrates that by mediating the underlying drivers, inherent conditions can strongly influence the buffer capacity of nonlinear systems.

## 1. Introduction

Identifying where critical thresholds occur in systems characterized by nonlinear dynamics is fundamental to objectively quantifying their resilience [1–3]. Ecosystems as diverse as freshwater lakes, grasslands, coral reefs, and macroalgal communities show hysteretic behaviour [4]; after collapse, these systems may not recover their initial state, even when stress conditions abate. These altered states are typically maintained by increases in the abundance of key species that reinforce stabilizing feedback [5]. There is growing recognition of external stressors as triggers of state shifts, and an increasing interest in understanding their underlying mechanisms [6–9]. Several critical state-changing agents have been identified, including overfishing, pollution and abnormal nutrient loading, population outbreaks of grazers, and infrequent disturbances like storms, fires, temperature anomalies, and other stochastic events [2,10]. Among the best described of these shifts occurs when herbivorous sea urchins, released from predation, quickly overtake near-shore macrophyte communities (i.e. kelp forests), reducing them to functionally depauperate barrens [11]. These altered states are maintained by feedback that prevents recovery even when herbivores are subsequently controlled. While in most ecosystems these drivers are relatively well understood, accurately predicting where critical transitions lie is not trivial. How systems respond to state-changing stressors may differ considerably, dependent on context-specific conditions. The difficulty of controlling all potential stressors limits most studies to examining the effects of a single major driver [6]. In reality though, a host of other apparently insignificant factors, acting together, may be critical in predisposing ecosystems to shifts. These factors may often vary intrinsically between locations.

The inherent conditions that determine resilience include a range of structuring environmental and ecological regimes (rainfall or fire regimes, natural nutrient loads, hydrodynamics, temperature, etc.) [4,10,12]. These may interact in complex ways with ecosystem processes, mediating stabilizing feedback and the mechanisms that trigger system shifts. For instance, under intense grazing pressure, Sahelian grasslands shift from perennial grasses either to annual grasses (capable of fairly rapid recovery) or shrubs (an altered stable state). Which trajectory the system takes is dictated by rainfall regimes, with drought conditions predisposing hysteretic shifts to a shrub-dominated assemblage [13]. Similarly, the resilience of many marine systems (coral reefs, kelp forests, etc.) can be strongly mediated by natural nutrient regimes [14,15]; post-collapse recovery is significantly retarded when nutrients facilitate recruitment, growth, and space-occupation of competitors [16–18]. These conditions can vary considerably with locations, making ecosystem trajectories intrinsically difficult to predict [16,17].

Reducing uncertainty in complex systems requires a better handle on how context-specific underlying conditions modify ecosystem processes. Attempts to anticipate thresholds have focused on examining characteristics of boundary conditions as signals of impending change [1,2,18,19]. These may manifest as subtle changes in the variance and skew of key system variables, self-organized patchiness, or a slowing down in ecosystem dynamics [20–23]. These changes in system behaviour serve as powerful early-warning indicators presaging major state shifts—either catastrophically [19] or more continuously [24]. Typically, signals have been derived from ecosystem models or by hind-casting of systems that have already experienced shifts. Finding meaningful predictive metrics that work in real-world situations is still elusive. These indicators are essentially phenomenological and dependent on long-term monitoring [19]. Their advantage is that they provide transcendent insights on system behaviour applicable across systems. However, they are not geared to illuminating specific mechanisms underlying transitional responses for a particular ecosystem. Identifying these causal mechanisms may require acknowledging that several interdependent biotic and abiotic processes drive the functioning of the system. Understanding these mechanisms would allow for a clearer evaluation of ecosystem resilience, providing managers with unambiguous directions in prioritizing ameliorative measures.

**Figure 1.**
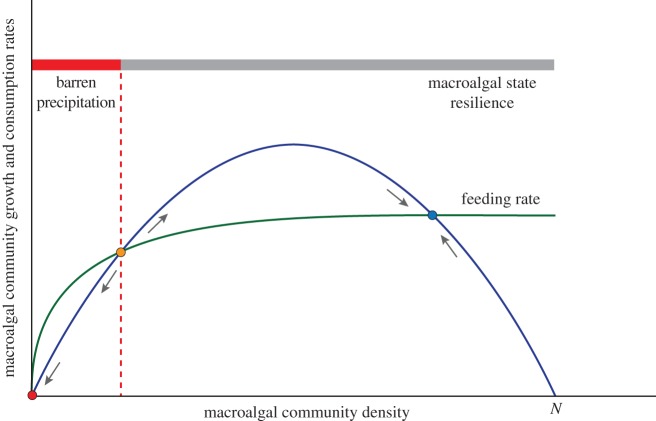
A macroalgal community grows up to a carrying capacity *N* following the solid blue line in the absence of herbivores. A population of herbivores consumes this macroalgae community at a rate represented by the solid green line (intermediate pressure, May 1977). Under this consumption curve, two stable states exist; a barren state (red point) and a well-structured macroalgal state (blue point). One unstable state exists (orange point) in which situations on the left precipitate barren formation (consumption > growth) and situations on the right enhance the macroalgal community stability (consumption < growth). The distance between the unstable point and red points represents the barren precipitation state and the distance between the unstable point and the carrying capacity *N* represents the macroalgal state resilience.

Mediterranean rocky macroalgal communities are useful models to explore how regional conditions may mediate ecosystem transitions: they show nonlinear responses, are relatively simple, and occur in conditions that differ considerably in their inherent nutrient regimes [11,25,26]. Overfished, these systems often shift to urchin-dominated barrens when their populations cross critical thresholds [11,26,27] (herbivory rates surpass plant growth (figure 1)). We examined if inherent nutrient regimes mediate where thresholds occur in response to urchin biomass [25]. Additionally, we examined potential mechanistic pathways by which nutrients modify these thresholds. We hypothesize that nutrient regimes can determine the relationship between growth rate and consumption by (i) influencing herbivore consumption rates based on food quality and/or (ii) modifying macroalgal growth based on nutrient availability. Given these mechanisms, macroalgal communities in low-nutrient regimes will experience sudden shifts to barrens at lower urchin biomasses than in high-nutrient regimes. We used complementary approaches to test these mechanisms including empirical observations and controlled laboratory and field experiments. We developed a simple consumer–resource model incorporating these mechanisms to explore how nutrients mediate ecosystem state changes.

## 2. Material and methods

### (a) Study system and principal objectives

Shallow Mediterranean rocky shores are dominated by macroalgae, constituting a very structured and diverse community [28,29]. Although growing in oligotrophic conditions, nutrient regimes vary considerably between regions: western continental shores typically receive high-nutrient inputs (i.e. riverine), while the eastern Mediterranean and islands are comparatively nutrient poor [30]. Together with the fish, *Sarpa salpa*, *Paracentrotus lividus* is among the most important herbivores in Western Mediterranean macroalgal communities, precipitating regime shifts when outbreaks occur [26,31]. Predator overfishing releases urchin populations from predation pressure, and macroalgal systems give way to barrens under sustained herbivory [26,32]. Once created, several reinforcing feedbacks maintain this new state. These include enhanced post-settlement survival of sea urchins, reduced potential for algal recruitment, reductions in the recruitment of predatory fish, and the facilitation of other herbivores [33–35]. We (i) used observational studies to establish the relationship between nutrient levels and critical transitions in macrophyte communities, (ii) identified the mechanisms underlying these transitions to understand how contrasting nutrient regimes influence resilience, and (iii) built a predictive model to explain how these transitions vary with inherent conditions. First, we examined how algal cover changed along a gradient of urchin biomass (from macroalgal forests to barrens) in regions with different nutrient conditions. For the second objective, we assessed two potential mechanisms underlying nutrient-mediated thresholds: (i) herbivores respond to nutrient impoverishment by changing their feeding rates and/or (ii) plant growth is facilitated by nutrients. Finally, we triangulated these results with a predictive consumer–resource model to understand how nutrients influence threshold conditions. This triangulated approach helped build confidence in the overall results.

#### (i) Determining thresholds under different nutrient regimes

To determine if nutrient regimes influence ecosystem thresholds, we surveyed shallow macroalgal rocky communities in two regions within the Mediterranean Sea characterized by different nutrient conditions (i.e. Catalan coast in Spain—high nutrients—and Sardinia Island in Italy—low nutrients; [30]). We confirmed these differences by measuring nutrient content in *Posidonia oceanica* seagrass shoots (a reliable indicator of nutrient availability [36]) at each location. The closest seagrass meadow was selected, usually within tens of metres of the macroalgal community. We collected five shoots when possible and used leaf tissue for nutrient analyses. At two sites, shoots were pooled together because the tissue was not enough for nitrogen analyses. For the rest of the sites the data obtained was averaged for each location. Data were analysed by comparing nitrogen content between regions where locations represented replicates. Results confirmed that the selected regions differed considerably in nutrients with a 26% difference in nitrogen content between high-nutrient and low-nutrient regimes (electronic supplementary material, figure S3; *p*-value < 0.03). These differences match with previous studies and patterns observable from satellite imagery (electronic supplementary material, figures S1 and S2*a*). In other respects, the two regions shared similar environmental features (sea surface temperature, exposure, light availability, salinity approximately 37.5 ppt etc.; electronic supplementary material, figure S2). To assess the relationship between urchin biomass and algal cover, we selected four localities per region (electronic supplementary material, figure S1) characterized by a similar assemblage of benthic algae (photophilic structuring algae of the genus *Dictyota, Halopteris* and *Padina* among others). Locations within each region were separate enough (tens to hundreds of kilometres) to avoid pseudoreplication (electronic supplementary material, figure S1).

At each locality, we sampled the substrate (approx. 3 m depth) using 50 × 50 cm quadrats at different urchin densities (60 quadrats per site when possible; *n* = 237 for the high-nutrient region, *n* = 185 for the low-nutrient region). We estimated total algal cover (capped at 100% of turf and canopy-forming species) as a measure of community state. As urchins can sometimes hide between algae or inside crevices, algal cover was measured after removing urchins from quadrats when necessary (i.e. when large urchins were present) to avoid underestimating algal cover. To assess grazing pressure, we counted all urchins (*P. lividus* and *A. lixula*) within each quadrat, classifying them into size-based age classes (post-settlers, less than 1 cm test diameter, TD; juveniles, 1–3 cm TD; young adults, 3–5 cm TD and adults, more than 5 cm TD). Size classes were used to calculate urchin biomass (wet weight, g m^−2^) using standard volumetric conversions for these species [37]. Only urchins larger than 3 cm were used to assess thresholds because smaller urchins contribute negligibly to grazing. We used total urchin biomass as a comparative metric of grazing pressure between regions because it integrates urchin size and accounts for herbivory. The principal species across all the localities was *P. lividus* (60–80% of total biomass).

Our observations indicated a threshold in the response of algal cover to urchin biomass. We examined this with change point detection methods using the package strucchange [1,38] in R [39] for each region. The algorithm assesses whether different parts of datasets require different fits to a linear regression. To assess the significance of every potential change point, an *F*-statistic (Chow test) was computed. This test is typically used in time-series for systems exposed to disturbances, but can be applied to detect abrupt changes in many other datasets [1]. As the change point detection requires discrete cover values we used mean percentage cover per urchin biomass.

#### (ii) Mechanisms underlying the thresholds

We explored mechanisms underlying nutrient-mediated thresholds using a combination of controlled laboratory and field experiments.

(*i*) *Feeding response.* In the laboratory, we tested if macroalgal nutrient content affected *per capita* urchin herbivory rates. We used the Mediterranean seaweed *Cystoseira mediterranea* as a model forage species because it is clearly preferred by *P. lividus* [31]. Sea urchins and macroalgae were collected in the same area (41.7° N 2.8° E). Half the macroalgae was fertilized (F) in aquaria (approx. 10 l) with running seawater for 3 days using 6 g of fertiliser (12N : 8P : 16 K) while the other non-fertilized (NF) were kept in natural seawater aquaria. To assess food quality, we measured leaf nitrogen (%N) from algal fronds from each treatment (F and NF, *n* = 5 per treatment). Fronds were powdered and analysed for nitrogen concentration. Fertilization successfully increased the nutrient content by approximately 9% (electronic supplementary material, figure S2; *p*-value < 0.01). All collected urchins were starved for 3 days in a holding aquarium (approx. 1 000 l). They were then transferred to six independent aquaria for testing. Each aquarium was divided into six compartments. Five of these accommodated a single urchin, and one was left without urchins (*n* = 30). We fed half the urchins with 4 g (wet weight) of the non-fertilized algae while the rest were fed with 4 g of the fertilized algae (*n* = 15 urchins per treatment). Compartments left without urchins, served as procedural controls to account for non-feeding-related algal losses. After 6 days we weighed the remaining algae and consumption was estimated by subtracting the final from the initial weight and dividing by total feeding time (6 days). No significant change in fertilized or unfertilized algal wet weight was detected in procedural controls. We used a linear mixed effects model to test for differences in consumption rates between fertilized and unfertilized treatments. The full model included the dependent variable ‘consumption rate’, the fixed factor ‘treatment’ (two levels: fertilized and unfertilized), and the random effect ‘aquarium’ (six levels–the six aquaria) to account for the variance among animals kept within the same aquarium. Each effect was dropped sequentially, and we selected the best model using the Akaike Information Criterion [40].

(*ii*) *Nutrient-mediated algal growth.* To measure how nutrients influenced algal growth in field conditions, we used two bare rock areas (completely overgrazed areas, 0% algal cover), one in each nutrient region. There, three herbivore exclusion cages of 50 × 50 cm (2 cm mesh size) were established. We measured changes in total algal cover (of both erect and turf algae) inside each plot after one month in both high- and low-nutrient conditions. We used change in algal cover as an indirect, comparative index of growth [41,42]. We measured algal cover using the same methods employed in our field surveys (see above). We used one-way ANOVAs to test for differences in growth using R [39]. Prior to the analysis, assumptions of normality and homogeneity of variances were checked both visually and statistically (i.e. Shapiro test and Bartlett test, respectively).

(*iii*) *Feeding response and nutrient-mediated growth* (*field experiment*). To further validate these mechanisms, we conducted a manipulative experiment to independently estimate the effects of nutrient-mediated growth and feeding response in real-world conditions. The experiment was conducted in an oligotrophic location (41.0° N 9.0° E) at approximately 8 m depth in a landscape dominated by isolated boulders during the maximum vegetative growth period for most algal species in the study area (May to September). Twelve isolated boulders (approx. 2 m^2^ in area) were randomly selected and used as independent plots (more than 5 m apart) in a total area of about 1 000 m^2^. All urchins were removed and algae scraped off with a metal brush to ensure similar initial conditions. Treatments were randomly assigned to plots: three fertilized (nutrient-enriched, without urchins: N_+_U_0_), three fertilized and had urchins added at a density of 10 individuals m^−2^ (nutrient-enriched, with urchins: N_+_U_+_), three unfertilized and had sea urchins added at a density of 10 individuals m^−2^ (not enriched, with urchins N_0_U_+_) and finally, three left unfertilized and without urchins (not enriched, without urchins: N_0_U_0_). Enriched conditions were achieved by fixing 20 × 5 × 5 cm nylon mesh bags (1 mm mesh size), containing 150 g fertilizing pellets (15N : 9P : 9 K) to a tile. The effectiveness of fertilization was assessed from water samples collected at randomly selected plots. Samples were taken approximately 1 cm from the nutrient source, filtered (0.45 µm filter size) and analysed using a continuous-flow AA3 Auto-Analyzer to determine the seawater nitrate concentration (mg l^−1^). Fertilization was always successful: N concentrations in fertilized plots were about 98% higher compared with unfertilized ones (*p* < 0.01). The number of urchins in U_+_ treatments was periodically controlled [33]. Algal cover on the boulders was estimated after six weeks using four random photographic quadrats (400 cm^2^ in size) of the surface of each plot. Nutrient-mediated growth in the absence of herbivory was calculated as the percentage difference in cover between fertilized and unfertilized treatments:2.1
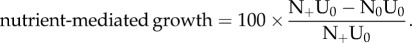
Urchin addition treatments integrated the effects of both nutrient-mediated growth and feeding response:2.2
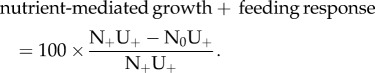
This allowed us to estimate the effect of compensatory feeding alone, by subtracting the effects of nutrient-mediated growth (equation (2.1)) from the total unconsumed algae (equation (2.2))
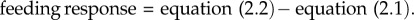


#### (iii) Modelling nutrient-mediated thresholds

We developed a simple consumer–resource model incorporating the mechanisms of nutrient-mediated growth and compensatory feeding to predict differences in tipping points. In the model, algal cover was introduced as a logistic growth curve while the effects of urchin herbivory were modelled as a Holling Type II equation. As a consequence, the interplay between algal demographics and urchin herbivory determined a dynamic equilibrium for algal cover through time. In this model, nutrients influenced both algal growth rates and urchin herbivory—the second by mediating handling time within the Holling Type II Equation (see electronic supplementary material, appendix A).

## 3. Results

### (a) Determining thresholds under different nutrient regimes

**Figure 2.**
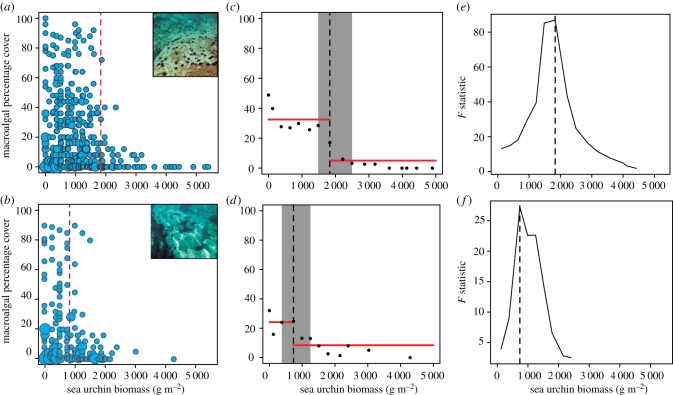
Bubble plots of the percentage of algal cover for different sea urchin biomass for the two regions sampled: (*a*) Catalan coast (*n* = 237) and (*b*) Sardinia Island (*n* = 185). Bubble size is proportional to the number of times a specific combination of urchin biomass and macroalgal cover was recorded. Dashed red lines indicate the position of thresholds. Photos are representative of typical barren areas in each region. Change point analysis results for high- (*c*) and low- (*d*) nutrient regions. Black dots show the mean algal cover at different levels of the stressor (urchin biomass in grams per square metre of wet weight). Red horizontal lines represent the mean algal cover before and after the threshold. Grey areas represent the confidence intervals (95%) around change points detected. The *F* statistic (Chow test) obtained for the forward threshold is presented for both high- (*e*) and low-nutrient regions (*f*). *F* statistic peaks around the area where the change point has been detected.

There was clear evidence that the macroalgal systems we sampled showed alternate states in both high- and low-nutrient regions (figure 2). Sudden changes in the community state (per cent cover) occurred when the stressor (urchin biomass) crossed critical values (tipping point). In high-nutrient conditions, results show that this tipping point was breached at urchin biomasses more than twice (figure 2*a*) that of low-nutrient conditions (figure 2*b*). Threshold analyses confirmed the existence of regime shifts in both nutrient regimes; thresholds were found at significantly different levels of the stressor (urchin biomass; figure 2*c,d*) in different nutrient conditions. Macroalgal communities in low-nutrient conditions shifted abruptly to urchin barrens when stressor values crossed 736 g m^−2^ sea urchin biomass (approx. 20 sea urchins m^−2^ of 5 cm TD, 380–1 250 g m^−2^, 95% confidence intervals; figure 2*d,f*). By contrast, in high-nutrient conditions canopy-forming algae were still present at biomasses around 1 832 g m^−2^ (approx. 40 sea urchins m^−2^, 1 484–2 494 g m^−2^, 95% confidence intervals; figure 2*c,e*); beyond this level however, these systems also collapse to urchin-dominated barrens.

### (b) Mechanisms underlying thresholds

**Figure 3.**
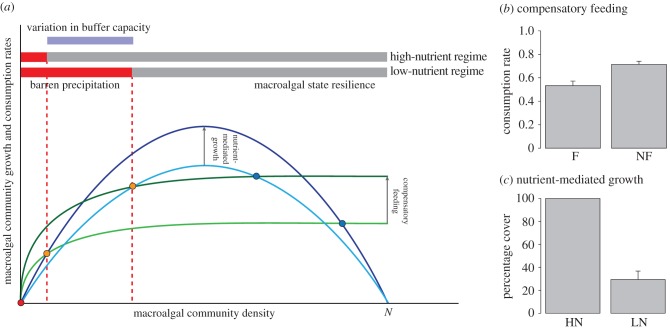
Conceptual diagram describing the two mechanisms evaluated; the compensatory feeding and nutrient-mediated growth of the macroalgal community and sea urchin population described in figure 1*a*. Under low-nutrient regimes sea urchins compensate feeding (i.e. increased consumption rates) (*b*) mean consumption rate on fertilized *C. mediterranea* *F* = 0.53 + 0.04 g day^−1^ (*n* = 15) and on non-fertilized *C. mediterranea* NF = 0.71 + 0.03 g day^−1^ (*n* = 15) in laboratory experiments after 6 days. At the same time under low-nutrient regimes, the macroalgal community presents retarded growth rates, (*c*) mean percentage cover of erect and turf algae in the high-nutrient region HN = 100% + 0% and low nutrient region LN = 29.3% + 7.42% after one month of herbivore exclusion.

Urchins showed significant foraging plasticity, adapting to lower nutrient conditions by increasing feeding rates (compensatory feeding). In the laboratory, urchin grazing rates were 25% higher when offered non-fertilized *C. mediterranea* compared with fertilized algae (figure 3*b*; *p* < 0.01; electronic supplementary material, figure S4). In field experiments, urchins in unfertilized plots ate considerably more than in fertilized plots. After accounting for nutrient-mediated growth, compensatory feeding amounted to 35.5% of total consumption in the N_0_U_+_ plots compared to N_+_U_+_ plots (electronic supplementary material, figure S3).

In addition, nutrients strongly influenced the growth of algae—growing significantly more in nutrient-rich regimes as well as in fertilized plots. After a month of herbivore exclusion, barren sites in both regions were recolonized by a very similar assemblage of mixed turfs and erect algae. However, while in the high-nutrient regime, caged sites recovered 100% of their algal cover, in the low-nutrient regime, only approximately 30% of the substrate was recolonized (figure 3*c*; *p* < 0.01; electronic supplementary material, figure S4). Fertilization experiments confirmed these results; fertilized plots (N_+_U_0_) showed a 35% increase in algal cover compared to controls (N_0_U_0_) (electronic supplementary material, S3).

### (c) Modelling nutrient-mediated thresholds

**Figure 4.**
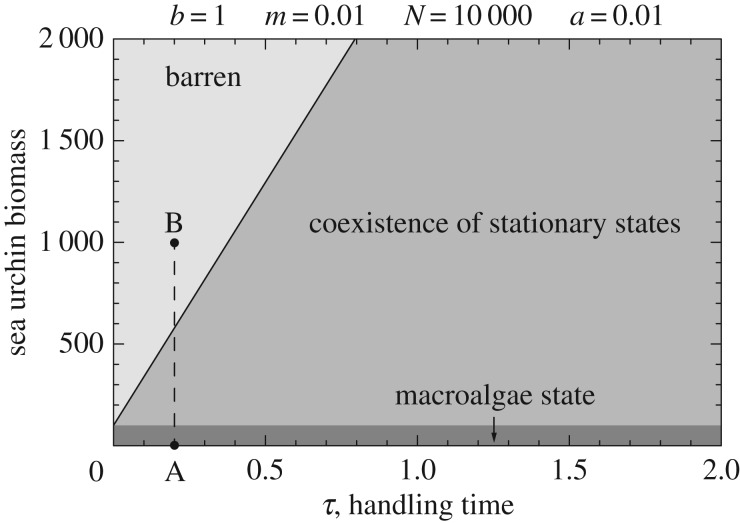
Under ‘rapid searching regime’, the area of the parameter space where regime shifts are prone to occur is relative large. The range of urchin densities that allows for coexistence of two stable stationary states increases as handling time increases. Parameter values are given above and have been chosen to satisfy the ‘rapid searching condition’ 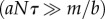, and match the range of sea urchin densities found in the field (see electronic supplementary material, appendix A). A gradient of sea urchin pressure (from A to B) takes the system from a macroalgae-dominated state (*a*) to a barren state (*b*).

**Figure 5.**
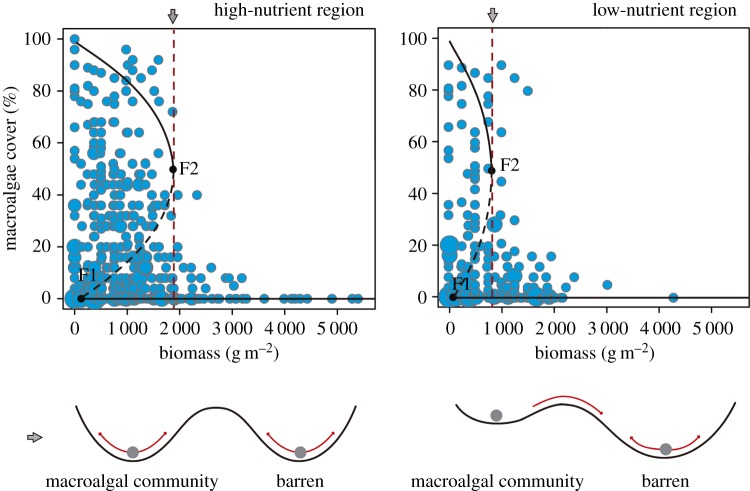
Buffer capacity of macroalgal community and barrens in each nutrient regime. The bubble graph shows macroalgal percentage cover related to sea urchin biomass in gram per metre square (wet weight) in the two regions. The dashed red line represents the threshold after a tipping point F2 is reached. F1 represents the tipping point from which the recovery of the macroalgal state is possible. Both F1 and F2 are set in the position determined by the consumer–resource mathematical model (see electronic supplementary material, appendix A). The schematic drawing below shows the resilience of each stable state in both nutrient regimes. The valleys represent the alternative stable states and the depth of the valley represents the resilience of that particular state.

The consumer–resource model identified clear bistability in this system (figure 4), with macroalgae rapidly shifting to barrens beyond a threshold. This threshold varied with nutrient conditions, governed largely by differences in growth rates and handling time. Additionally, as forage encounter rates are typically high compared with algal growth rates, it requires urchin populations to reduce very close to zero before the barren is likely to shift back to algal forests (figure 5, electronic supplementary material; appendix A). This was true for both nutrient regimes. This matches closely with the empirical data and, when parametrized with ecologically meaningful values, predicts thresholds very close to those observed in the field.

## 4. Discussion

While nonlinear dynamics of temperate macrophyte communities has long been recognized, identifying where nonlinearities lie has resisted prediction. Our results indicate that nutrient regimes can strongly determine the buffer capacity of macroalgal communities, mediating the amount of herbivory the system can withstand. Results from field, laboratory, and modelling approaches, all converged to give us a clear understanding of how nutrients mediate the grazing resilience of these near-shore benthic communities. Specifically, oligotrophic regions are much less resilient to grazing compared with nutrient-rich regions (figure 5). We documented a clear shift to barrens at around half the sea urchin biomass in oligotrophic systems (relative to high-nutrient areas), indicating that they are intrinsically less able to cope with grazing. Barren states in both nutrient regimes were remarkably stable. Our model highlights the existence of hysteresis indicating that it would require an almost-complete disappearance of sea urchins before these systems recover their macroalgal state. This is consistent with other studies [43,44]. We identified the mechanisms underlying differences in these shifts, showing that both compensatory grazing and reduced algal growth act together in oligotrophic systems, endowing them with roughly half the buffer capacity of more nutrient-rich systems (figures 3 and 5). Understanding and quantifying how context-specific conditions influence threshold dynamics will take us one step closer towards reducing the inevitable surprise of nonlinear ecological systems.

Temperate macrophyte communities appear particularly prone to catastrophic shifts [11,26], with sea urchin overgrazing (linked to population outbreaks) being the primary trigger of these events. While it is uncertain from our work what drives differences in urchin populations, studies have highlighted the importance of both supply-side processes governing recruitment and settlement [45] as well as subsequent top-down control by fish predators [26,46–48] in driving urchin population dynamics. Temperate macroagal systems show catastrophic state-shifting behaviour independently of local and regional conditions. We documented sudden shifts to urchin barrens in both nutrient regimes, linked clearly to increases in urchin abundance. The difference was in where thresholds lay, with relatively nutrient-rich systems more than twice as resilient to grazing compared with nutrient-poor systems. Given their susceptibility to discontinuities, determining these boundary conditions is all the more important to manage temperate macrophyte communities in functionally healthy states. This is critical given that our model shows strong hysteretic behaviour, an indicator of the resilience of the altered community states [11,25]. While low-nutrient conditions make macrophyte systems much more prone to barrens, recovery from this state appears to be independent of nutrient regimes (figure 4, electronic supplementary material; appendix A). The model suggests that as long as algal encounter rates are much higher than algal regrowth, recovery from barrens will always be very protracted. While the backward process needs to be interpreted with caution, it indicates that erect algae are likely to recover only when urchin abundances reach close to zero, regardless of nutrient conditions. Once barrens are created, urchin populations generally do not collapse although individual growth rates may decrease as they switch to feeding less nutritious encrusting algae [25,44]. In macroalgal-barren systems, urchins continue scraping the substrate, maintaining areas free of algae. In addition, other barren-associated biota may also play significant roles in enhancing the stability of barrens [49]. Such reinforcing feedbacks make recovery difficult, emphasizing the need to prevent collapses from occurring. Being able to accurately predict transitions is essential for preventive action to be effective.

Our findings contribute to understanding the role environmental factors play in determining the resilience of macroalgal-dominated reefs. Wernberg *et al.* recently described the collapse of kelp forests following a severe heatwave [9]. Additionally, Vergés empirically demonstrated how warming-mediated increases in fish herbivory trigger system collapses [50]. These recent examples highlight the urgent need to uncover the mechanisms underlying the worldwide decline of temperate macroalgal reefs [51]. Our work shows that inherent conditions can be critical drivers of buffer capacity by influencing both plant growth and herbivore feeding responses. Hence, very oligotrophic systems may be particularly vulnerable to herbivore outbreaks. Cardona *et al.* [52] described how, after a pulse in productivity in a low-nutrient region, the abundance of urchins increased, resulting in dramatic reductions in macroalgal communities. In the eastern Mediterranean, with significantly lower nutrient conditions than the western basin [30], the spread of the herbivorous fish *Siganus* spp. through the Suez Canal has led to extreme depletions of canopy-forming algae and, where well-developed macrophyte communities were once dominant, now bare rock prevails [29,53]. Acknowledging this differential vulnerability may require designing context-specific strategies for managing these systems based on measurable differences in their inherent buffer capacity.

Our work also explores the potential mechanisms that can explain the differential resilience of these ecosystems. Under relatively nutrient-poor conditions, macroalgae showed clearly reduced rates of growth and urchins offset the low quality of plants by increasing their feeding rates to meet nutritional requirements [54]. Both compensatory feeding and nutrient-mediated growth work together (figure 4) making macroalgal systems in low-nutrient regimes shift to barrens at much lower herbivore levels compared with communities in high-nutrient conditions. The underlying nutrient regime determines the degree to which macroalgal growth can support urchin consumption before collapsing. As we demonstrate, low-nutrient regimes increased rates of consumption by herbivores (compensatory feeding, figure 3*b*; electronic supplementary material, figure S5) while simultaneously reducing the growth capacity of macroalgae (figure 3*c*; electronic supplementary material, figure S5). This maximized grazing:primary production ratio caused a faster shift to a new macroalgae-free urchin barren (figure 3). These overshoots are much more likely to occur in the characteristic nutrient-poor conditions of islands making them much less resilient to herbivory compared with other nutrient-rich systems. Under extremes of high-nutrient conditions the situation may change. In these scenarios (relatively rare in the Mediterranean), macroalgal community composition may change to fast-growing species, making the system vulnerable to collapse through a completely different set of mechanisms including shading and overgrowth [55]. Based on our current results however, we suspect that oligotrophic systems may follow inherently different trajectories than non-oligotrophic systems, and need to be addressed separately.

Several studies have focused on determining signals of impending collapse in nonlinear systems [2,19,23]. They identify potentially useful proxies (critical slowing down, increasing variance and skewness, etc.) that may herald approaching thresholds. Identifying these signals depends on reliable long-term monitoring, and adequate demonstration that signals correlate with hysteretic change. There have been few real-world examples where these leading indicators have been able to predict imminent collapse in time for ameliorative action. Essentially phenomenological, these approaches assume that the underlying mechanisms will vary from context to context. Our work indicates that, where it is possible to unpack these underlying drivers, it can help substantially in identifying where and why tipping points occur. Additionally, clarifying the mechanisms that govern these dynamics allows determining the role inherent conditions play in mediating critical thresholds. Taken together, it provides a way forward to make regime-specific predictions of buffer capacity of systems at local to regional scales. These are admittedly much more difficult to establish in more complex systems where multiple mechanisms act in several synergistic and antagonistic ways. Our contention is that, even in these more complex ecosystems, inherent conditions may predispose the system to very different dynamics, implying very different ecosystem responses. It is critical to shift attention to a more mechanistic understanding of the ecological processes that govern nonlinear systems. Determining how feedbacks interact with context-specific conditions will help considerably improve the predictive power of resilience models, reducing the surprise inherent in identifying thresholds and improving our ability to manage systems characterized by nonlinear behaviours.

## Supplementary Material

Map of the study locations

## Supplementary Material

Map of Mediterranean environmental characteristics

## Supplementary Material

P. oceanica nutrient content

## Supplementary Material

C. mediterranean nutrient content

## Supplementary Material

Feeding response and nutrient mediated growth

## Supplementary Material

Appendix A

## Data accessibility

Data from surveys are stored in the open access Temperate Reef Base (http://temperatereefbase.imas.utas.edu.au/static/landing.html) and Data deposited in Dryad: http://dx.doi.org/10.5061/dryad.7k300 [56].

## References

[1] Andersen T, Carstensen J, Hernández-García E, Duarte CM 2009 Ecological thresholds and regime shifts: approaches to identification. Trends Ecol. Evol. 24, 49–57. (10.1016/j.tree.2008.07.014) 18952317

[2] Scheffer M, Carpenter S 2003 Catastrophic regime shifts in ecosystems: linking theory to observation. Trends Ecol. Evolut. 18, 648–656. (10.1016/j.tree.2003.09.002)

[3] May RM 1977 Thresholds and breakpoints in ecosystems with a multiplicity of stable states. Nature 269, 471–477.

[4] Scheffer M, Carpenter S, Foley JA, Folke C, Walker B 2001 Catastrophic shifts in ecosystems. Nature 413, 591–596. 11595939 10.1038/35098000

[5] Petraitis PS, Dudgeon SR 2004 Detection of alternative stable states in marine communities. J. Exp. Mar. Biol. Ecol. 300, 343–371. (10.1016/j.jembe.2003.12.026)

[6] Conversi A et al. 2015 A holistic view of marine regime shifts. Phil. Trans. R. Soc. B 370, 20130279 (10.1098/rstb.2013.0279)

[7] Terborgh J et al. 2001 Ecological meltdown in predator-free forest fragments. Science 294, 1923–1926. (10.1126/science.1064397) 11729317

[8] Estes JA et al. 2011 Trophic downgrading of planet Earth. Science 333, 301–306. (10.1126/science.1205106) 21764740

[9] Wernberg T et al. 2016 Climate-driven regime shift of a temperate marine ecosystem. Science 353, 169–172. (10.1126/science.aad8745) 27387951

[10] Folke C, Carpenter S, Walker B, Scheffer M, Elmqvist T, Gunderson L, Holling CS 2004 Regime shifts, resilience, and biodiversity in ecosystem management. Annu. Rev. Ecol. Evol. Syst. 35, 557–581. (10.2307/annurev.ecolsys.35.021103.30000021)

[11] Filbee-Dexter K, Scheibling RE 2014 Sea urchin barrens as alternative stable states of collapsed kelp ecosystems. Mar. Ecol. Prog. Ser. 495, 1–25. (10.3354/meps10573)

[12] Wernberg T, Thomsen MS, Tuya F, Kendrick GA, Staehr PA, Toohey BD 2010 Decreasing resilience of kelp beds along a latitudinal temperature gradient: potential implications for a warmer future. Ecol. Lett. 13, 685–694. (10.1111/j.1461-0248.2010.01466.x) 20412279

[13] Rietkerk M, Ketner P, Stroosnijder L, Prins HH 1996 Sahelian rangeland development; a catastrophe? J. Range. Manage. 49, 512–519.

[14] Connell SD, Irving A 2009 The subtidal ecology of rocky coasts: Local-regional-biogeographic patterns and their experimental analysis. In Marine Macroecology, pp. 392–418. Chicago, IL: University of Chicago Press.

[15] Wiedenmann J, D'Angelo C, Smith EG, Hunt AN, Legiret FE, Postle AD, Achterberg EP 2012 Nutrient enrichment can increase the susceptibility of reef corals to bleaching. Nat. Clim. Change 3, 160–164. (10.1038/nclimate1661)

[16] Holling CS 1973 Resilience and stability of ecological systems. Annu. Rev. Ecol. Evol. Syst. 4, 1–23.

[17] Gorman D, Russell BD, Connell SD 2009 Land-to-sea connectivity: linking human-derived terrestrial subsidies to subtidal habitat change on open rocky coasts. Ecol. Appl. 19, 1114–1126. (10.1890/08-0831.1) 19688920

[18] Hastings A, Wysham DB 2010 Regime shifts in ecological systems can occur with no warning. Ecol. Lett. 13, 464–472. (10.1111/j.1461-0248.2010.01439.x) 20148928

[19] Scheffer M et al. 2009 Early-warning signals for critical transitions. Nature 461, 53–59. (10.1038/nature08227) 19727193

[20] Carpenter S, Brock WA 2006 Rising variance: a leading indicator of ecological transition. Ecol. Lett. 9, 311–318. (10.1111/j.1461-0248.2005.00877.x) 16958897

[21] Guttal V, Jayaprakash C 2008 Changing skewness: an early warning signal of regime shifts in ecosystems. Ecol. Lett. 11, 450–460. (10.1111/j.1461-0248.2008.01160.x) 18279354

[22] Rietkerk M, Dekker SC, de Ruiter PC, de Koppel van J 2004 Self-organized patchiness and catastrophic shifts in ecosystems. Science 305, 1926–1929. 15448261 10.1126/science.1101867

[23] Dakos V, Carpenter S, van Nes EH, Scheffer M 2015 Resilience indicators: prospects and limitations for early warnings of regime shifts. Phil. Trans. R. Soc. B 370, 20130263 (10.1098/rstb.2013.0263)

[24] Kéfi S, Dakos V, Scheffer M, van Nes EH, Rietkerk M 2012 Early warning signals also precede non-catastrophic transitions. Oikos 122, 641–648. (10.1111/j.1600-0706.2012.20838.x)

[25] Ling SD et al. 2015 Global regime shift dynamics of catastrophic sea urchin overgrazing. Phil. Trans. R. Soc. B 370, 20130269 (10.1098/rstb.2013.0269)

[26] Pinnegar JK, Polunin NVC, Francour P, Badalamenti F 2000 Trophic cascades in benthic marine ecosystems: lessons for fisheries and protected-area management. Environ. Conserv. 27, 179–200.

[27] Sala E, Zabala M 1996 Fish predation and the structure of the sea urchin *Paracentrotus lividus* populations in the NW Mediterranean. Mar. Ecol. Prog. Ser. 140, 71–81. (10.3354/meps140071)

[28] Sales M, Ballesteros E 2009 Shallow *Cystoseira* (Fucales: Ochrophyta) assemblages thriving in sheltered areas from Menorca (NW Mediterranean): relationships with environmental factors and anthropogenic pressures. Estuar. Coast Shelf S 84, 476–482. (10.1016/j.ecss.2009.07.013)

[29] Sala E et al. 2012 The structure of Mediterranean Rocky Reef ecosystems across environmental and human gradients, and conservation implications. PLoS ONE 7, e32742 (10.1371/journal.pone.0032742.t004) 22393445 PMC3290621

[30] Bosc E, Bricaud A, Antoine D 2004 Seasonal and interannual variability in algal biomass and primary production in the Mediterranean Sea, as derived from 4 years of SeaWiFS observations. Glob. Biogeochem. Cy. 18, GB1005 (10.1029/2003GB002034)

[31] Boudouresque C-F, Verlaque M 2001 Ecology of Paracentrotus lividus. Amsterdam, The Netherlands: Elsevier.

[32] Boada J, Arthur R, Farina S, Santana Y, Mascaró O, Romero J, Alcoverro T 2015 Hotspots of predation persist outside marine reserves in the historically fished Mediterranean Sea. Biol. Cons. 191, 67–74. (10.1016/j.biocon.2015.06.017)

[33] Bulleri F, Benedetti-Cecchi L, Cinelli F 1999 Grazing by the sea urchins *Arbacia lixula* L. and *Paracentrotus lividus* Lam. in the Northwest Mediterranean. J. Exp. Mar. Biol. Ecol. 241, 81–95. (10.1016/S0022-0981(99)00073-8) 11090849

[34] Guidetti P, Fraschetti S, Terlizzi A, Boero F 2003 Distribution patterns of sea urchins and barrens in shallow Mediterranean rocky reefs impacted by the illegal fishery of the rock-boring mollusc Lithophaga lithophaga. Mar. Biol. 143, 1135–1142. (10.1007/s00227-003-1163-z)

[35] Cheminee A, Sala E, Pastor J, Bodilis P, Thiriet P, Mangialajo L, Cottalorda J-M, Francour P 2013 Nursery value of Cystoseira forests for Mediterranean rocky reef fishes. J. Exp. Mar. Biol. Ecol. 442, 70–79. (10.1016/j.jembe.2013.02.003)

[36] Roca G, Alcoverro T, de Torres M, Manzanera M, Martínez-Crego B, Bennett S, Farina S, Pérez M, Romero J 2015 Detecting water quality improvement along the Catalan coast (Spain) using stress-specific biochemical seagrass indicators. Ecol. Indic. 54, 161–170. (10.1016/j.ecolind.2015.02.031)

[37] Ballesteros E 1981 Algunos datos biométricos de *Paracentrotus lividus* (Lmk.), *Arbacia lixula* (L.) y *Sphaerechinus granularis* (Lmk.) (Echinodermata, Echinoidea). Oecol. Aquat. 5, 227–231.

[38] Zeileis A, Leisch F, Hornik K, Kleiber C 2013 An R Package for Testing for Structural Change in Linear Regression Models. 1–17.

[39] R Development Core Team. 2013 R: A language and environment for statistical computing. Vienna, Austria See http://R-project.org/.

[40] Zuur A, Ieno EN, Walker N, Saveliev AA, Smith GM 2009 Mixed effects models and extensions in ecology with R. Berlin, Germany: Springer.

[41] Sala E, Kizilkaya Z, Yildirim D, Ballesteros E 2011 Alien marine fishes deplete algal biomass in the Eastern Mediterranean. PLoS ONE 6, e17356 (10.1371/journal.pone.0017356) 21364943 PMC3043076

[42] Ballesteros E 1991 Homage to Ramon Margalef; or, Why there is such pleasure in studying nature (eds JD Ros, N Prat) Oecologia Aquatica 10, 223–242.

[43] Ling SD, Johnson CR, Frusher SD, Ridgway KR 2009 Overfishing reduces resilience of kelp beds to climate-driven catastrophic phase shift. Proc. Natl Acad. Sci. USA 106, 22 341–22 345. (10.1073/pnas.0907529106) PMC2793314 20018706

[44] Hill NA, Blount C, Poore AGB, Worthington D, Steinberg PD 2003 Grazing effects of the sea urchin Centrostephanus rodgersii in two contrasting rocky reef habitats: effects of urchin density and its implications for the fishery. Mar. Freshw. Res. 54, 691–700. (10.1071/MF03052)

[45] Prado P, Tomas F, Pinna S, Farina S, Roca G, Ceccherelli G, Romero J, Alcoverro T 2012 Habitat and scale shape the demographic fate of the keystone sea urchin *Paracentrotus lividus* in Mediterranean macrophyte communities. PLoS ONE 7, e35170 (10.1371/journal.pone.0035170.t002) 22536355 PMC3335053

[46] Guidetti P 2004 Consumers of sea urchins, *Paracentrotus lividus* and *Arbacia lixula*, in shallow Mediterranean rocky reefs. Helgol. Mar. Res. 58, 110–116. (10.1007/s10152-004-0176-4)

[47] Sala E 1997 Fish predators and scavengers of the sea urchin *Paracentrotus lividus* in protected areas of the north-west Mediterranean Sea. Mar. Biol. 129, 531–539. (10.1007/s002270050194)

[48] Guidetti P 2006 Marine reserves reestablish lost predatory interactions and cause community changes in rocky reefs. Ecol. Appl. 16, 963–976. 16826995 10.1890/1051-0761(2006)016[0963:mrrlpi]2.0.co;2

[49] Piazzi L, Bulleri F, Ceccherelli G 2016 Limpets compensate sea urchin decline and enhance the stability of rocky subtidal barrens. Mar. Environ. Res. 115, 49–55. (10.1016/j.marenvres.2016.01.009) 26874891

[50] Vergés A et al. 2016 Long-term empirical evidence of ocean warming leading to tropicalization of fish communities, increased herbivory, and loss of kelp. Proc. Natl Acad. Sci. USA 113, 13 791–13 796. (10.1073/pnas.1610725113) PMC5137712 27849585

[51] Krumhansl KA et al. 2016 Global patterns of kelp forest change over the past half-century. Proc Natl Acad. Sci. USA 113, 13 785–13 790. (10.1073/pnas.1606102113) PMC5137772 27849580

[52] Cardona L, Moranta J, Reñones O, Hereu B 2013 Pulses of phytoplanktonic productivity may enhance sea urchin abundance and induce state shifts in Mediterranean rocky reefs. Estuar. Coast. Shelf S 133, 88–96. (10.1016/j.ecss.2013.08.020)

[53] Vergés A et al. 2014 The tropicalization of temperate marine ecosystems: climate-mediated changes in herbivory and community phase shifts. Proc. R. Soc. B 281, 20140846 (10.1098/rspb.2014.0846) PMC4100510 25009065

[54] Valentine JF, Heck KL Jr 2001 The role of leaf nitrogen content in determining turtlegrass (*Thalassia testudinum*) grazing by a generalized herbivore in the northeastern Gulf of Mexico. J. Exp. Mar. Biol. Ecol. 258, 65–86. (10.1016/S0022-0981(00)00342-7) 11239626

[55] Moy FE, Christie H 2012 Large-scale shift from sugar kelp (*Saccharina latissima*) to ephemeral algae along the south and west coast of Norway. Mar. Bio. Res. 8, 309–321. (10.1080/17451000.2011.637561)

[56] Boada J et al. 2017 Immanent conditions determine imminent collapses: nutrient regimes define the resilience of macroalgal communities. *Dryad Digital Repository*. (10.5061/dryad.7k300) PMC5378086 28330920

