# Supplementary figures and images for: Immanent conditions determine imminent collapses: nutrient regimes define the resilience of macroalgal communities
Source: Proc Biol Sci. 2017 Mar 22;284(1851):20162814. doi: 10.1098/rspb.2016.2814 (PMC5378086; doi:10.1098/rspb.2016.2814)

# Annual Productivity ( $\text{gC} \cdot \text{m}^{-2}$ )

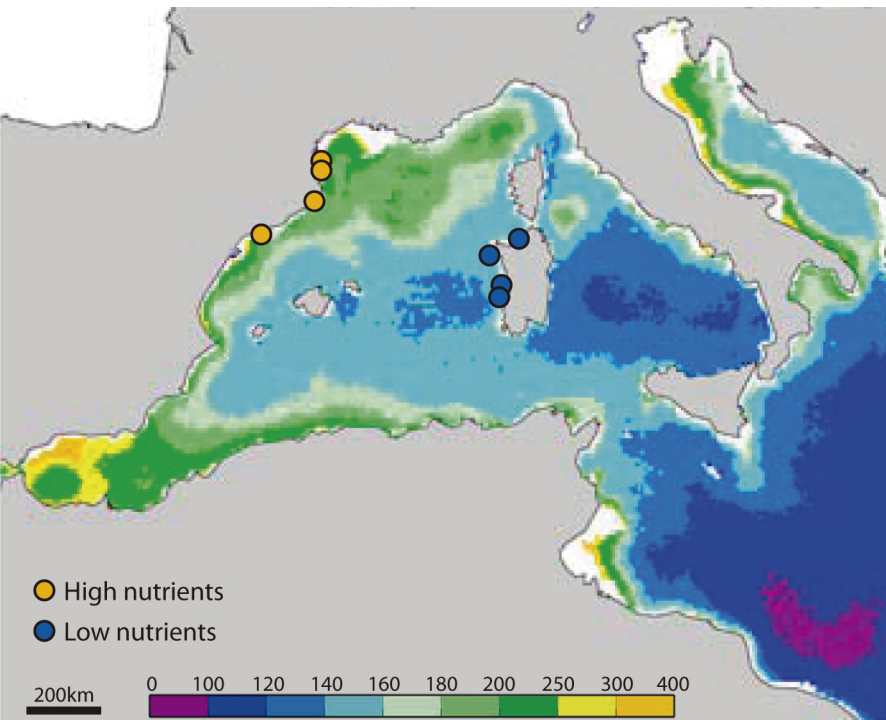

Supplement: Map of the study locations [file rspb20162814supp1.pdf]

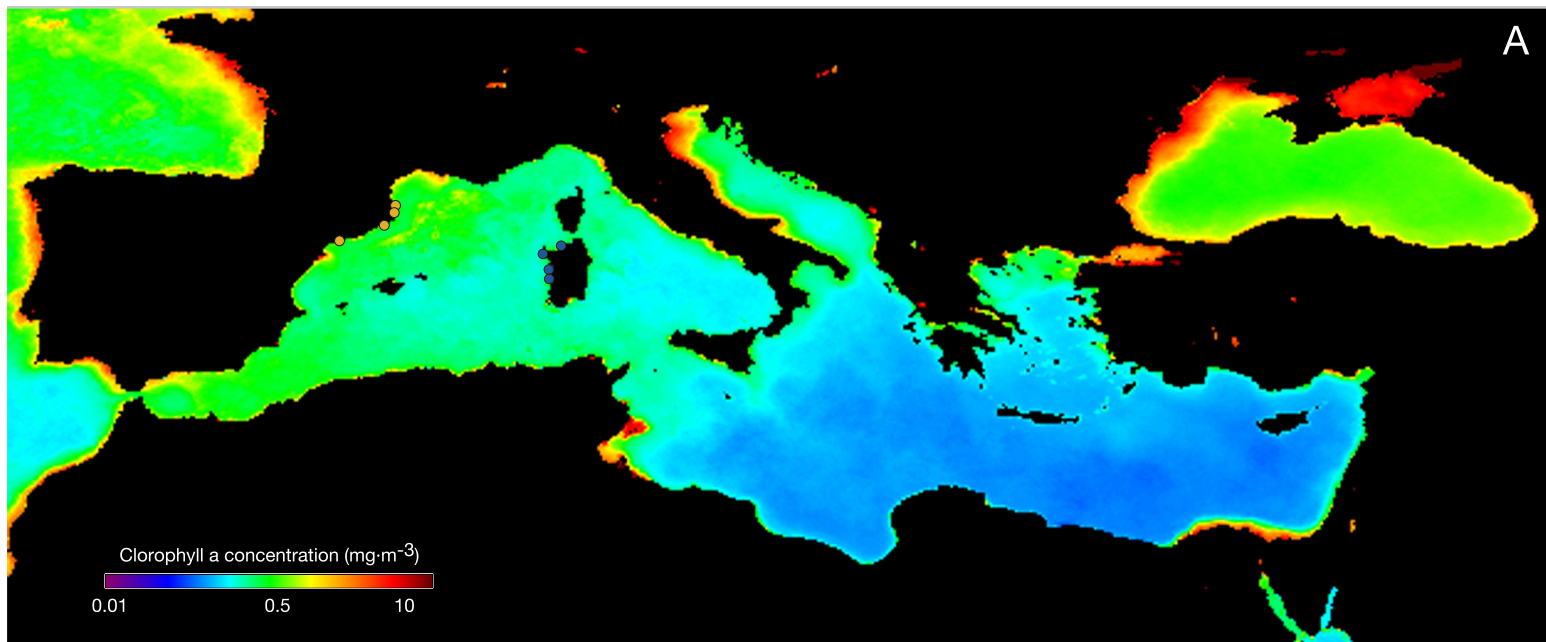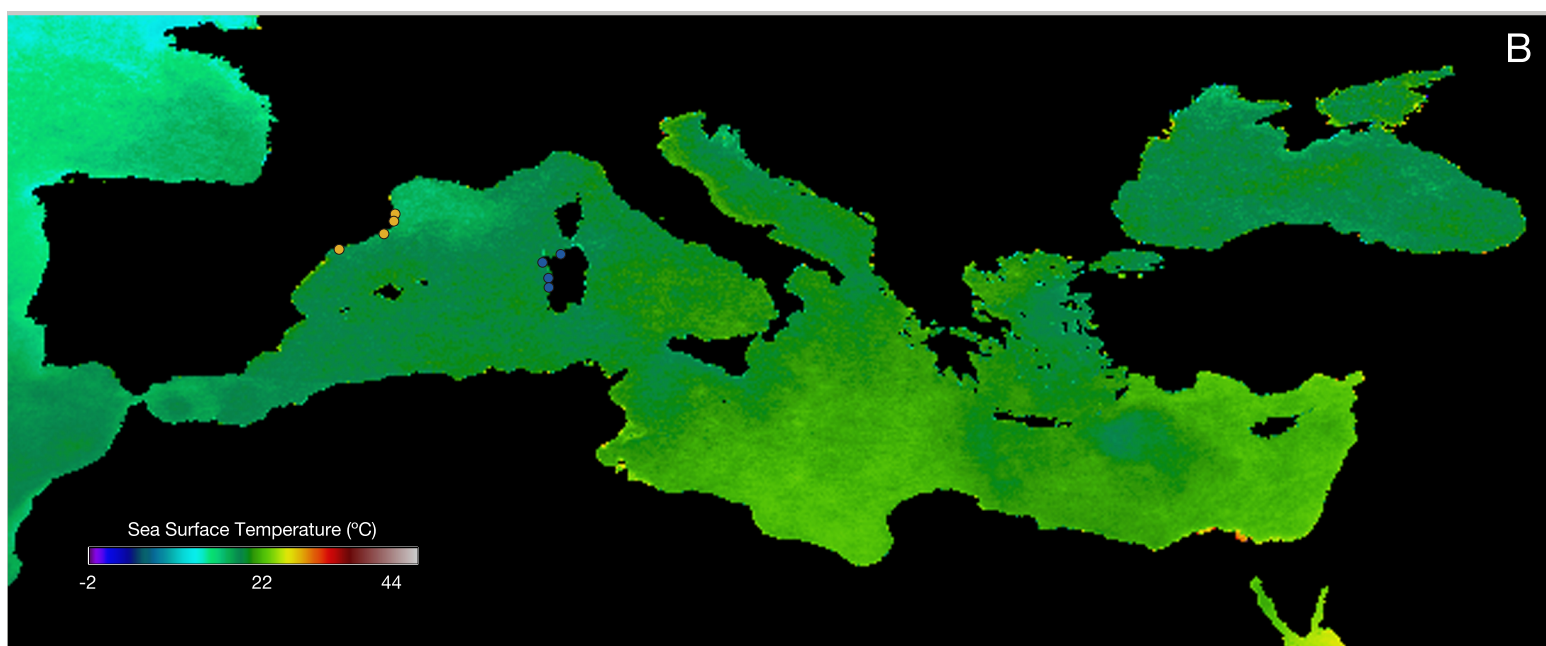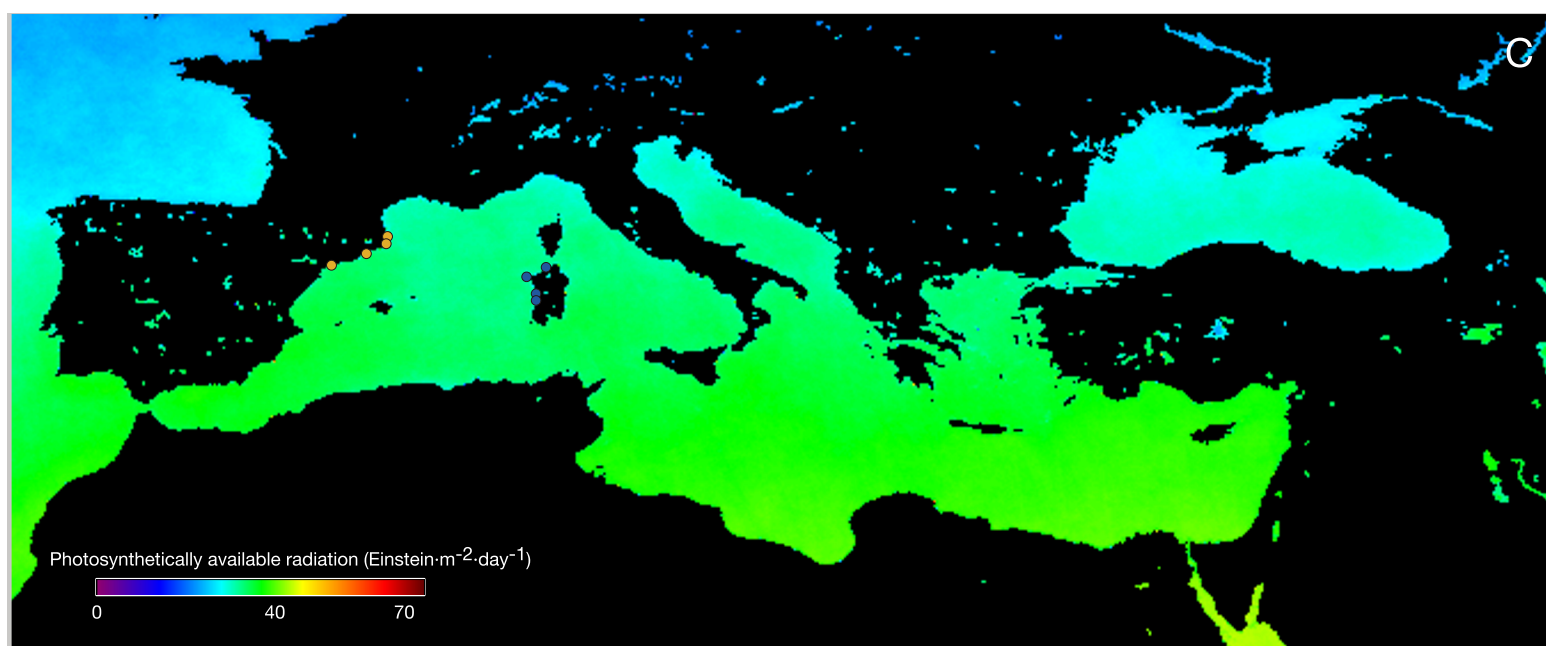

Supplement: Map of Mediterranean environmental characteristics [file rspb20162814supp2.pdf]

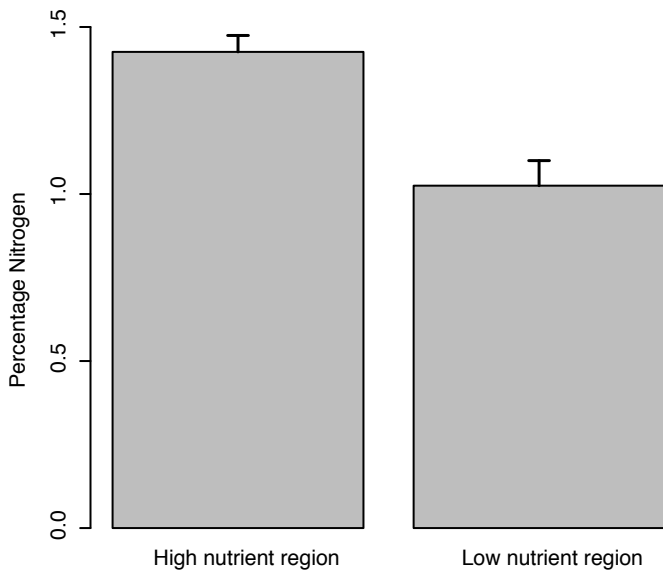

Supplement: P. oceanica nutrient content [file rspb20162814supp3.pdf]

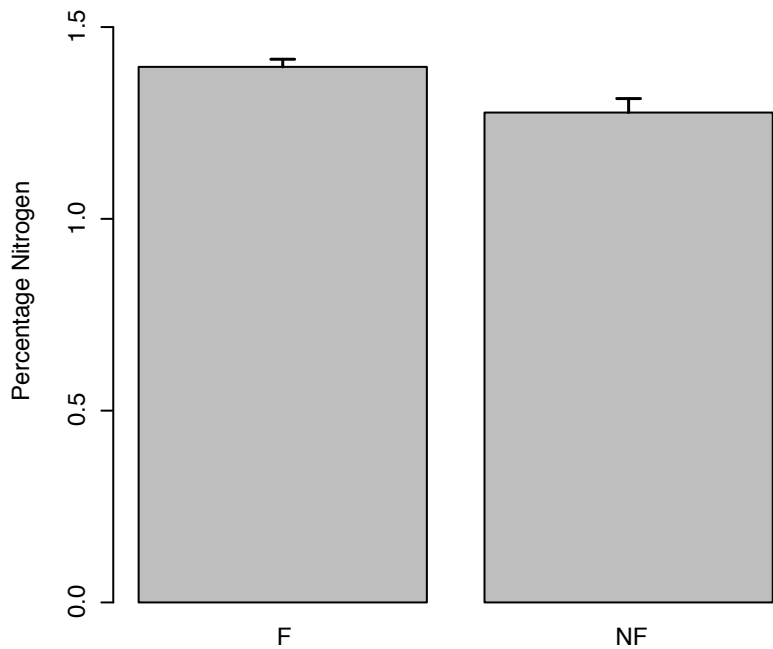

Supplement: C. mediterranean nutrient content [file rspb20162814supp4.pdf]

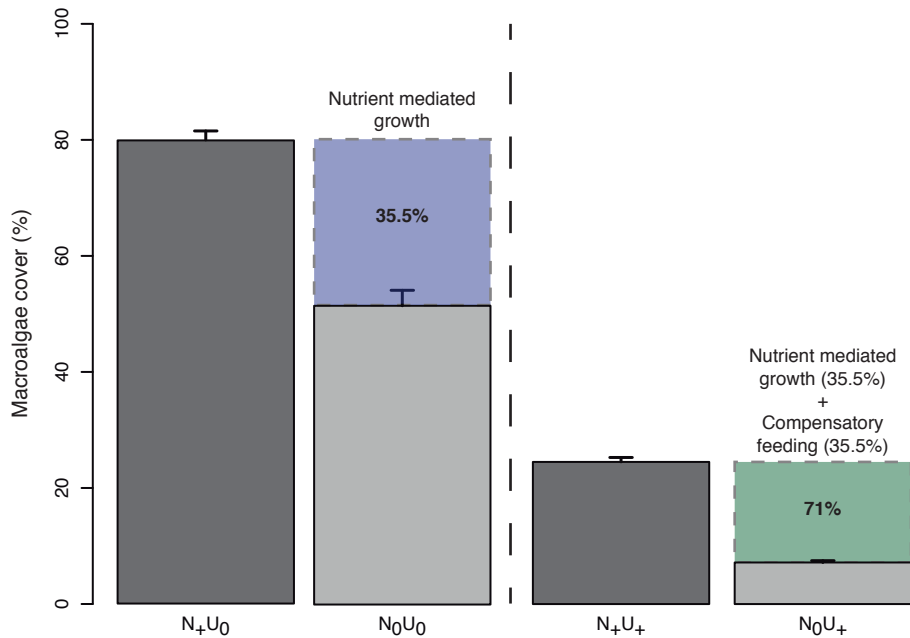

Supplement: Feeding response and nutrient mediated growth [file rspb20162814supp5.pdf]
