# Supplementary material for: Immanent conditions determine imminent collapses: nutrient regimes define the resilience of macroalgal communities
Source: Proc Biol Sci. 2017 Mar 22;284(1851):20162814. doi: 10.1098/rspb.2016.2814 (PMC5378086; doi:10.1098/rspb.2016.2814)
Supplement: Appendix A [file rspb20162814supp6.pdf]

Immanent conditions determine imminent collapses: nutrient regimes define the grazing resilience of macroalgal communities

Jordi Boada[1], Rohan Arthur[1,2], D. Alonso[1], Jordi Pagès[1,3], Albert Pessarrodona[1], Silvia Oliva[4], Giulia Ceccherelli[4], Luigi Piazzi[4], Javier Romero[5], and Teresa Alcoverro[1]

[1] Center for Advanced Studies (CEAB-CSIC), Spanish Council for Scientific Research, Blanes, Spain.
[2] Nature Conservation Foundation, 4th Cross, Gokulam Park Road, Mysore, India
[3] School of Ocean Sciences, Bangor University, Menai Bridge, Wales, LL59 5AB, United Kingdom
[4] Dipartimento di Scienze della Natura e del Territorio (DIPNET), Università di Sassari. Via Piandanna 4, Sassari, Italy
[5] Departament d'Ecologia, Facultat de Biologia, Universitat de Barcelona. Av. Diagonal, 643. 08028 Barcelona, Spain

Appendix A

The growth of macroalgae on a rocky surface is usually logistic: the algae grows asymptotically until it reaches certain percentage cover. At steady-state, the total surface covered by macroalgae will be determined by the interplay between perturbation regimes (death rates), and the ability of algae to recover (growth rates). If X represents the number of macroalgae stipes per unit area, N is the maximum number of stipes that can be physically packed per unit area, and b and m are the percapita birth and death rates, respectively, then the temporal evolution of macroalgae density towards asymptotic cover is described by the following equation:

$$\frac{dX}{dt} = bX(1 - \frac{X}{N}) - mX \tag{1}$$

It is well known [2] that the fraction of macroalgae cover at stationarity is given by:

$$p = 1 - \frac{m}{b} \tag{2}$$

where asymptotic cover, p, is defined as X/N.

A herbivore population, such as sea urchins, feeding on a logistically growing resource can be modeled using a Holling Type II functional response:

$$F(X; a, \tau) = \frac{aX}{1 + a\tau\, X} \tag{3}$$

where a and τ are encounter rates and handling times, respectively.

This function represents how percapita herbivore feeding rates respond to resource density. The analysis of this functional response shows that, as resource density increases, per capita

resource consumption rates tend to be only limited by herbivore handing time, τ. In fact, the asymptotic percapita feeding rate is given by $1/\tau$. Therefore, in principle, non-resource-limited herbivores feed at constant percapita rates:

$$f_\infty \equiv \lim_{X\to\infty} F(X; a, \tau) = \frac{1}{\tau} \tag{4}$$

However, it is reasonable to assume that this asymptotic herbivore behavior is influenced by the nutrient quality of the resource as urchins respond with compensatory feeding. In nutrient-rich environments, asymptotic feeding rates probably decrease, because herbivores can incorporate the same quantity of nutrients in shorter times. If we assume that nutrient requirements are metabolically controlled, required herbivore nutrient intake per unit time can be written as the product of resource feeding rates —food consumption per unit time— times the nutrient concentration of that food, a measure of food quality. Therefore, for non-resource-limited herbivores, this is, at high food densities, asymptotic percapita feeding rates and food nutrient content should be hyperbolically related ($K = f_\infty\, n$):

$$f_\infty = \frac{K}{n} \tag{5}$$

where n is the nutrient content of macroalgae per unit resource biomass, which can be written, inversely, in terms of herbivore handling times:

$$\tau = \frac{n}{K} \tag{6}$$

To sum up, under compensatory feeding, this argument shows that handling times should increase as nutrient content per unit macroalgae biomass increases, this is, in nutrient-rich environments.

When herbivore dynamics of reproduction and death is very slow compared to herbivore resource consumption and the inherent dynamics of the resource, it is useful to assume constant herbivore densities affecting resource mortality rates through consumption. Using a Holling Type II functional response, the temporal evolution of resource density can be described by the following equation:

$$\frac{dX}{dt} = bX(1 - \frac{X}{N}) - mX - \frac{a\,Y}{1 + a\tau\, X}X \tag{7}$$

where Y is a constant population density.

In 1987, in the context of the importance of stochasticity and non-linear, non-average behavior, P.A. Allen [1] showed that this dynamics allows for the coexistence of stationary states and sudden regime shifts from resource-dominated to resource-depleted environments as herbivore densities increase (see Fig 1). When we analyze the stationary states of this equation in the usual way, we conclude that there is always a stationary state at zero resource densities and, in addition, the possibility of two other states, which are given by:

$$p^\star_\pm = \frac{1}{2}\left(1 - \frac{1 + \frac{m}{b}aT}{aT}\right) \pm \frac{1}{2}\sqrt{\left(1 + \frac{1 - \frac{m}{b}aT}{aT}\right)^2 - 4\frac{Y}{bT}} \tag{8}$$

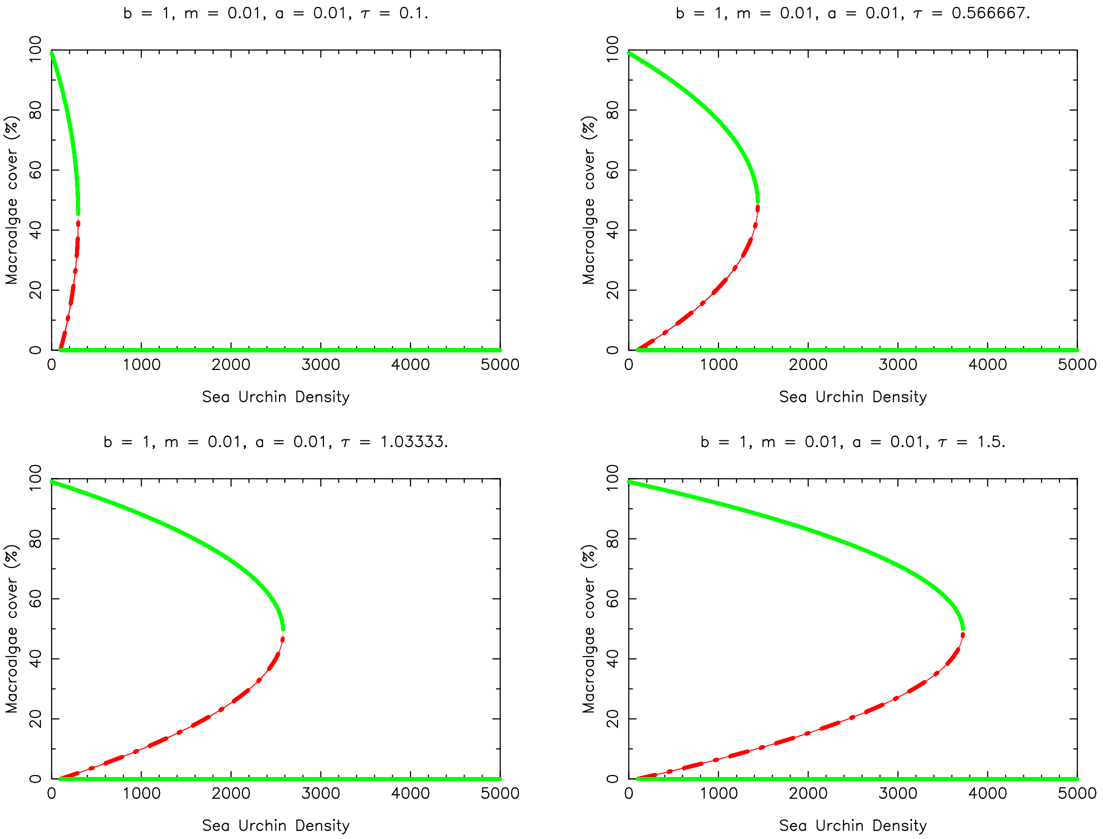

Figure 1. The range of urchin densities that allows for coexistence of two stable stationary states (curves in green) increases as handling time increases from the upper-left to the bottom-right panel. The lower stable branch almost reaches zero sea urchin density. Parameter values are given in the titles, but $N = 10000$. Their values have been chosen to satisfy the "rapid searching condition" ($aN\tau \gg m/b$), and approximately match the range of urchin densities found in the field.

where $p \equiv X/N$ and $T \equiv \tau N$.

By inspecting these stationary solutions, we observe that when herbivore density lies between the thresholds Y_0 and Y_1, three stationary states coexist: a stable upper stationary state, p_+, an unstable intermediate stationary state, p_-, and, finally, a stable lower stationary state at zero resource density. Fortunately, these two thresholds can be calculated and are given by:

$$Y_0 = \frac{b}{a}\left(1 - \frac{m}{b}\right) \quad (9)$$

$$Y_1 = \frac{bT}{4}\left(1 + \frac{1 - \frac{m}{b}aT}{aT}\right)^2 \quad (10)$$

In particular, in the regime of rapid sea urchin searching dynamics, that is when encounter rates are very fast compared with macroalgae re-growth, the first of these threshold values is very close to zero ($Y_0 \sim 0$).

Interestingly, notice that when $aN\tau \gg m/b$, the second threshold, Y_1, shifts, almost linearly, to higher values as handling times increase (see Fig 1):

$$Y_1 \approx \frac{bT}{4}\left(1 - \frac{m}{b}\right)^2 \quad (11)$$

We think that the natural sea urchin-macroalgae interaction lies in this regime, which can be called "rapid searching regime". As you see, there is a clear interpretation of this condition. According to this analysis, if the number of potential encounters with macroalgae individuals in τ time units ($aN\tau$) per individual urchin in comparison to the number macroalgae potential recruits in the average life time span of a macroalgae individual (m/b) is very high, then the upper threshold over which macroalgal community collapses, Y_1, grows approximately linearly with handling time (see Eq.11), which, as argued above, should increase with macroalgae nutrient content. This prediction is, qualitatively, fully supported by empirical data and field observations (see main text).

The lower threshold, Y_0, does not depend on handling time, but on macroalgae growth and death rates, on one hand, and on the encounter rate a, on the other. This latter rate is related to urchin movement abilities. As you see, the faster sea urchins search, the larger a is and the lower this threshold lies. This analysis predicts that under the "rapid searching regime", this threshold is very close to zero urchin densities. In addition, differences in between-site perturbation regimes (m) and algae growth conditions (b) introduces unavoidable variability in this threshold value. Taking together, and for these two reasons, we think the empirical estimation of the threshold Y_0 may be quite difficult. In any case, our theory predicts, under the "rapid searching regime", that the recovery of the marcroalgal community from the barren situation requires the removal of most urchins, in agreement with field observations and *in-situ* experiments (see main text).

References

1. P. M. Allen. Evolution: Why the whole is greater than the sum of the parts. In W. Wolf and C.-J. Soeder adn F. R. Drepper, editors, *Ecodynamics: Contributions to Theoretical Ecology*, Berlin, 1987. Springer-Verlag.

2. R. Levins. Some demographic and genetic consequences of environmental heterogeneity for biological control. *Bull. Entomol. Soc. Amer.*, 15:227–240, 1969.
